# Effective and Efficient Stand Magnifier Use in Visually Impaired Children

**DOI:** 10.3389/fpsyg.2016.00944

**Published:** 2016-06-23

**Authors:** Joyce Liebrand-Schurink, Ralf F. A. Cox, Ger H. M. B. van Rens, Antonius H. N. Cillessen, Ruud G. J. Meulenbroek, Frouke N. Boonstra

**Affiliations:** ^1^Behavioural Science Institute, Radboud UniversityNijmegen, Netherlands; ^2^Bartiméus Institute for the Visually ImpairedZeist, Netherlands; ^3^Expertise Center Health, Social Care and Technology, Saxion University of Applied SciencesEnschede, Netherlands; ^4^Department of Psychology, University of GroningenGroningen, Netherlands; ^5^VU University Medical CenterAmsterdam, Netherlands; ^6^Department Cognitive Neuroscience, Donders Institute for Brain, Cognition and Behaviour, Radboud University Medical CentreNijmegen, Netherlands

**Keywords:** visually impaired children, magnifier, fine motor skills, motor development, perceptuomotor task, low vision

## Abstract

**Purpose:** The main objective of this study was to analyze the effectiveness and efficiency of magnifier use in children with visual impairment who did not use a low vision aid earlier, in an ecologically valid goal-directed perceptuomotor task.

**Methods:** Participants were twenty-nine 4- to 8-year-old children with visual impairment and 47 age-matched children with normal vision. After seeing a first symbol (an Lea Hyvärinen [LH] symbol), children were instructed to (1) move the stand magnifier as quickly as possible toward a small target symbol (another LH symbol that could only be seen by using the magnifier), (2) compare the two symbols, and (3) move the magnifier to one of two response areas to indicate whether the two symbols were identical. Performance was measured in terms of accuracy, response time, identification time, and movement time. Viewing distance, as well as hand and eye dominance while using the magnifier was assessed.

**Results:** There were no significant differences between the two groups in accuracy, reaction time, and movement time. Contrary to the prediction, children with visual impairment required less time to identify small symbols than children with normal vision. Both within-subject and between-subject variability in viewing distance were smaller in the visually impaired group than in the normally sighted group. In the visually impaired group, a larger viewing distance was associated with shorter identification time, which in turn was associated with higher accuracy. In the normally sighted group, a faster movement with the magnifier and a faster identification were associated with increasing age.

**Conclusion:** The findings indicate that children with visual impairment can use the stand magnifier adequately and efficiently. The normally sighted children show an age-related development in movement time and identification time and show more variability in viewing distance, which is not found in visually impaired children. Visually impaired children seem to choose a standard but less adaptive strategy in which they primarily used their preferred hand to manipulate the magnifier and their preferred eye to identify the symbol.

**Trial registration:** Registered at http://www.trialregister.nl; NTR2380

## Introduction

For children with visual impairment, adequate use of a low vision aid (LVA) such as a magnifier is essential for everyday activities. Using an LVA has several advantages for visually impaired children (Cox et al., 2009; Schurink et al., 2011; Huurneman et al., 2013), but also demands complex behavior. There is a considerable gap in our understanding of LVA use in children and the specific problems and challenges they encounter. Previous research on LVA use has focused primarily on reading in adults. These studies provided valuable insights in, for example, the page navigation problem and oculomotor control (for a review, see Schurink et al., 2011). When children read a text with a magnifier, they see only a few characters at the time and must redirect the magnifier to incrementally process the characters forming a word, a process repeatedly occurring to read words (Beckmann and Legge, 1996). Children exploit visual information to direct the magnifier over the text (Phase 1) and at the same time exploit visual information for reading and understanding the text (Phase 2). The page navigation problem illustrates the alternation between Phases 1 and 2 during reading with a magnifier. When moving from word to word, the nature and relative influence of visual and control-related information constantly changes smoothly when action unfolds. At the end of a line, when the reader redirects the magnifier to the beginning of the next line, the “balance” changes abruptly from visually related to control-related information.

Efficient magnifier use requires motor skills, especially in Phase 1. The integration of sensory and motor systems is essential in the development of goal-directed action in infants (Ferronato et al., 2014) and children’s hand movements have not completed full maturation yet (Rueckriegel et al., 2008). Furthermore, children with visual impairment often show delayed motor development (Reynell, 1978; Sleeuwenhoek and Boter, 1995; Bouchard and Tetreault, 2000; Brambring, 2001; Celeste, 2002; Aki et al., 2008; Houwen et al., 2008; Reimer et al., 2008; Grant and Moseley, 2011; Lions et al., 2013, 2014) which might affect their ability to control the magnifier. The complexity of the task relates to the required level of motor and cognitive abilities (Schurink et al., 2011); a static task requires a lower level of motor and cognitive abilities than a dynamic task that entails simultaneous control of multiple action parameters. For example, research has shown that children with a developmental level of 2 years were capable of successfully performing a static magnifier task in which they had to name or match pictures and small objects with the use of a magnifier (Ritchie et al., 1989), whereas children older than 3.5 years could successfully perform a dynamic trail-following task in which they had to navigate the magnifier across a surface to follow a trail of symbols (Cox et al., 2007, 2009). In one study we examined motor control of an object that matched the size and shape of a stand magnifier, but did not provide magnification of any kind, we found that visually impaired children with infantile nystagmus syndrome, aged 4–8 years, performed slower, less accurate, and less efficient movements than normally sighted children (Liebrand-Schurink et al., 2015).

Efficient magnifier use requires perceptual skills such as visual information pick-up, accommodation, and monocular viewing to pick up the LVA-enlarged visual information, especially in Phase 2. The literature on children’s ability to efficiently use a magnifier is scarce. The visual system has considerable plasticity in infancy and childhood (Vedamurthy et al., 2008) and the effect of maturation on everyday LVA use is unknown. One study examined the use of a 90 mm diameter glass dome-magnifier with enlarged print in children with visual impairment (Huurneman et al., 2013). The investigators chose a magnifier with a large field of view and complete line coverage so that children did not need to move the magnifier and navigational demands could be excluded as a confounder. They concluded that a magnifier is equally effective as large print in improving the performance of these children on a near vision task (Huurneman et al., 2013).

The main objective of this study was to analyze the effectiveness and efficiency of magnifier use in an ecologically valid task (meaning that the task approximates real-life settings) in children with visual impairment who had no previous experience with a LVA. The children that participated were 4–8 years old. There are two reasons for choosing this age group. First, the introduction of a LVA early in a child’s life, around the age of 4, would be beneficial from a developmental perspective, because this is before children start to read, at this age children are less vulnerable to stigmatizing and it could partly prevent developmental delays (see Schurink et al., 2011). Second, the effect of maturation and development on everyday LVA use is unknown, therefore a wider age range was chosen. Visual impairment was defined as a visual acuity ≤0.4 (0.4 LogMAR) and ≥0.05 (1.30 Log MAR) in the better eye. The task consisted of identifying small symbols with a commonly used stand magnifier. The stand magnifier was chosen because it offers stable vision (Lee and Cho, 2007) and high magnification (6×) and can be manipulated with the entire hand. A sharp image can be attained by looking through the magnifier with one eye.

We expected both children with visual impairment and children with normal vision to perform the task with the magnifier *effectively*. We hypothesized that both groups would be equally successful, because symbol size was adjusted to the child’s visual acuity. Two hypotheses were tested regarding the *efficiency* of LVA use. First, we hypothesized that visually impaired children would need more time than normally sighted children in Phase 1, which primarily involves goal-directed arm movements with the LVA. This hypothesis was based on studies showing that fine and gross motor skills and goal-directed movements are less well developed in children with visual impairment than in children with normal vision (Reynell, 1978; Sleeuwenhoek and Boter, 1995; Bouchard and Tetreault, 2000; Brambring, 2001; Celeste, 2002; Aki et al., 2008; Houwen et al., 2008; Lions et al., 2013; Liebrand-Schurink et al., 2015) Second, we hypothesized that visually impaired children would need more time to identify a symbol under threshold than normally sighted children in Phase 2, because they have less experience with small details. Young children are used to accommodate when stimulated with details. This accommodative response is strong and is performed together with convergence (Bharadwaj and Candy, 2008). Binocular identification of details by accommodation and convergence is a normal response of young children who start to study tiny objects from the age of 1,5 or 2 years. However, visually impaired children appeared to be late in the development of this identification task. In a study with magnifier use in visually impaired children, we observed that most of these children needed more time to study small details while typically developing children do not need this time (Cox et al., 2009; Boonstra et al., 2012). In this study, both normally sighted and visually impaired children had no prior experience with LVA.

## Materials and Methods

### Participants

Participants were 29 children with visual impairment (*M*_age_ = 78 months; *M* visual acuity = 0.22 Snellen or 0.65 LogMAR) from client databases of Dutch vision rehabilitation centers and 47 children with normal sight (*M*_age_ = 79 months; *M* visual acuity = 1.00 or 0 LogMAR) from a regular primary school in Netherlands, aged 4–8 years. An ophthalmological exam was conducted to measure near and distance visual acuity, visual fields, and perception of contrast. Children were included if there were no known or reported intellectual and/or physical impairments, and if they had no previous LVA experience, normal birth weight (≥3000 g) and were born at term (≥36 weeks of gestation). Information regarding birth and the presence of additional impairments was obtained from (medical) records from either the school or the rehabilitation center. All children attended regular primary schools. Children with visual impairment were included if they had visual acuities between 0.4 (0.40 LogMAR) and 0.05 (1.30 LogMAR) in the better eye (E-chart, 6 m). Children with normal vision were included if they had visual acuities better than 0.8 (0.10 LogMAR). The study was approved by an accredited Medical Review Ethics Committee (CMO-Arnhem Nijmegen), and all protocols adhered to the guidelines of the Declaration of Helsinki. Informed consent was obtained from the parents of all children in the study.

### Ophthalmological Examination

**Table 1** shows the clinical details of the children with visual impairment. Distance visual acuity was measured monocularly and binocularly with correction with the Landolt C-test (Haase and Hohmann, 1982) at 5 m and the Illiterate E-chart (Taylor, 1978) at 6 m under controlled lighting conditions in an ophthalmological setting. Near-visual acuity (used to establish *M*-value threshold) was determined binocularly with the LH (Lea Hyvärinen) version of the C-test at 40 cm (Huurneman et al., 2012). Stereopsis was assessed with the Titmus Fly Test (Hasche et al., 2001), and if possible the TNO-test (Walraven, 1975; a red–green system). An orthoptic examination was performed by orthoptists who performed an alternate cover test, a cover–uncover test, and if necessary the four diopter base out prism test. A gross estimation of the visual field was obtained by confrontational techniques to secure full view at the digitizer tablet. Finally, a cycloplegia slit-lamp examination, funduscopy and objective refraction were obtained, and, if necessary, the spectacle correction was prescribed or changed. A new appointment was made for baseline measurement if a new correction was prescribed. All children with glasses wore them during the entire experiment.

**Table 1:** Clinical characteristics of children with visual impairment.

Child	Age (year)	DVA^†^		
		RE	LE	Binocular	NVA^‡^	Diagnosis	Refractive correction
1	4	0.8	0.8	0.9	0.8	Idiopathic INS	R: S: +0.25 C: -0.75 ax: 166 L: S: +0.50 C: -1.00 ax: 16
2	6	1.0	1.0	1.0	1.0	Achromatopsia, INS	R: S: +3.50 C: -3.50 ax: 8 L: S: +3.25 C: -2.50 ax: 174
3	4	1.4	1.4	1.4	1.2	Aniridia, INS	R: S:-4.75 C: -2.00 ax: 180 L: S: -4.5 C: -1.25 ax: 5
4	7	0.7	0.7	0.6	0.6	CSNB, INS	R: S: -3.75 C: -1.25 ax: 45 L: S: -3.50 C: -0.75 ax: 95
5	8	0.6	0.7	0.5	0.8	Idiopathic INS	No correction
6	5	1.0	1.2	0.9	0.9	Aniridia, INS	R: S: +3.75 C: -1.50 ax: 180 L: S: +3.25 C: -2.50 ax: 174
7	5	1.4	1.1	0.9	1.0	Albinism, INS	No correction
8	6	0.7	0.8	0.7	0.6	CSNB	R: S: -6.50 C: -1.50 ax: 176 L: S: -7.00 C: -1.50 ax: 155
9	8	0.5	1.0	0.4	0.4	Hypermetropia, INS	R: S: +0.50 C: -2.50 ax: 14 L: S: +1.00 C: -3.75 ax: 155
10	5	0.3	0.3	0.3	0.5	Cone dystrophy	R: S: -6.00 C: -1.00 ax: 2 L: S: -6.75 C: -0.75 ax: 50
11	5	1.1	1.1	1.0	1.2	Albinism, INS	R: S: +2.00C: -0.50 ax: 180 L: S: +3.75 C: -0.50 ax: 180
12	7	0.6	0.6	0.6	0.6	Idiopathic INS	R: S: +3.25 C: -1.25 ax: 8 L: S: +2.75 C: -1.25 ax: 180
13	5	1.1	1.1	1.0	1.1	Albinism, INS	R: S: +4.00 L: S: +4.00
14	4	0.6	0.5	0.6	0.5	CSNB	R: S: -7.25 C: -1.25 ax: 120 L: S: -7.25 C: -1.75 ax: 75
15	7	0.6	0.6	0.5	0.5	Ocular motility disorder	No correction
16	6	0.7	0.6	0.6	0.7	Myopia	R: S: -5.00 C: -3.25 ax: 2 L: S: -5.00 C: -3.25 ax: 1
17	5	0.8	0.8	0.7	0.8	Macular hypoplasia	R: S: +2.50 C: -2.00 ax: 177 L: S: +2.25 C: -1.75 ax: 7
18	5	0.6	nm	0.6	0.4	Idiopathic INS	No correction
19	5	0.8	0.8	0.7	0.7	Idiopathic INS	R: S: +2.00 C: -1 ax: 180 L: S: +2.00 C: -1 ax: 170
20	9	0.5	0.6	0.4	0.4	Albinism, INS	R: S: +1.50 C: -2.00 ax: 180 L: S: +1.50 C: -1.25 ax: 175
21	6	1.1	0.8	0.7	0.6	Congenital cataract (aphakia)	R: S: +2.5 C: -2.5 ax: 180 L: S: +2.5 C: -1.5 ax: 180
22	7	0.9	1.1	0.8	0.8	Albinism, INS	R: S: +0.50 C: -1.25 ax: 105 L: S: +plano C: -0.50 ax: 48
23	7	0.9	1.1	0.8	0.8	Idiopathic INS	R: S: +2.00 C: -1.25 ax: 172 L: S: +2.25 C: -0.50 ax: 6
24	8	0.4	0.4	0.4	0.3	Idiopathic INS	R: S: +4.50 L: S: +3.50 C: -0.50 ax: 180
25	6	0.8	1.0	0.5	0.5	Idiopathic INS	R: S +2.00 C: -1.5 ax: 130 L: S +3.00 C: -1.5 ax: 160
26	6	1.4	1.0	1.0	1.1	Albinism, INS	R: S: +4.25 C: -2.50 ax: 10 L: S: +4.75 C: -2.00 ax: 170
27	5	0.8	0.8	0.8	0.7	Albinism	R: S: +3.50 C: -1.25 ax: 177 L: S: +3.00 C: -1.25 ax: 172
28	7	1.1	1.1	0.8	0.9	Albinism, INS	R: S: +1.75 C: -2.00 ax: 5 L: S: +2.75 C: -2.50 ax: 172
29	5	0.8	0.7	0.7	0.8	Idiopathic INS	R: S: +1.00 L: S: +1.50

*Distance visual acuity of child number 10 was 0.3 LogMAR, but near visual acuity was 0.5 LogMAR and therefore the child was included in the study. ^†^Distance visual acuity (DVA) with C-test crowded version 2.6′ spacing at 5 m (LogMAR). ^‡^Near visual acuity (NVA) measured with C-test crowded version 2.6′ spacing at 40 cm (LogMAR). CSNB, congenital stationary night blindness; RE, right eye; LE, left eye; INS, infantile nystagmus syndrome.*

### Materials and Procedure

**FIGURE 1:**
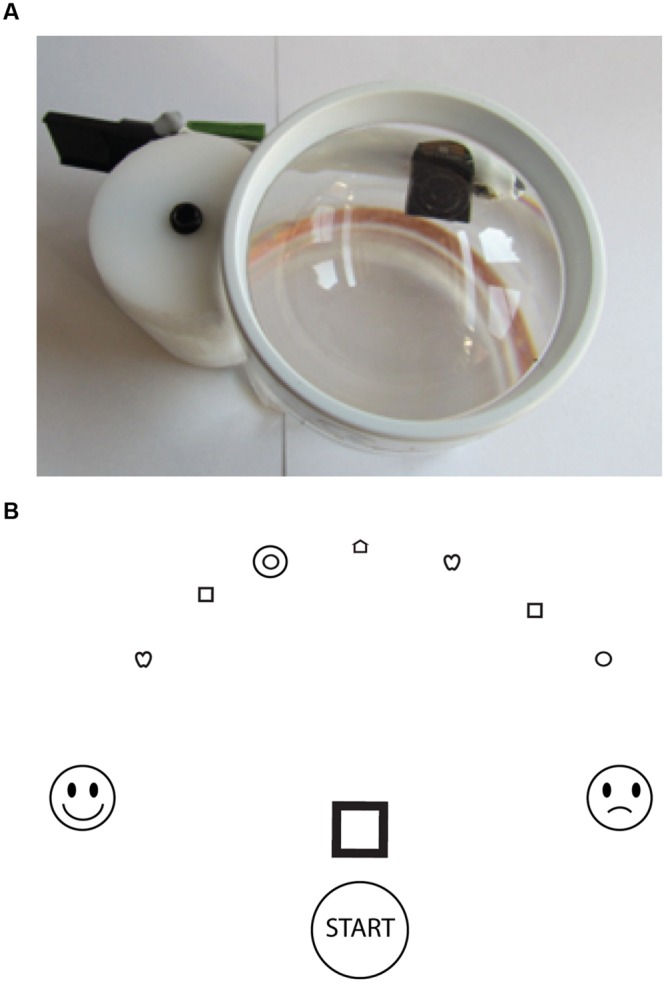
**(A)** The magnifier with the built-in camera and **(B)** an example of the information that was presented in the task. The start circle, large symbol (square), match-area (smiling icon), and non-match-area (sad icon) are displayed by the digitizer. The Lea Hyvärinen (LH) symbols three steps below threshold were printed on the sheet that was placed on top of the digitizer. During the experiment, one of the LH symbol was encircled (in this example the third symbol from the left) to indicate to which symbol the magnifier should be moved (target symbol).

The visual aid used in this study was a 23.0 diopter (aspheric lens) stand magnifier (Eschenbach, Nürnberg, Germany) with a magnification of six times, and an equivalent viewing distance of 4.3 cm. The magnifier is 48 mm in height and its lens housing has a diameter of 52 mm with a built-in camera (see **Figure 1A**). These dimensions make it suitable for young children to manipulate it with one or two hands. The magnifier is fit for monocular use, which is not always easy for young children (Bharadwaj and Candy, 2008). However, this magnifier was chosen to ensure that the subjects were unable to see the characters without the use of the magnifier. During task performance, the children were allowed to choose their own distance, because the task was supposed to be ecologically valid and should resemble an everyday situation in which children could chose their own strategy. They chose a distance of about 5–10 cm. which represents a magnification of about 10/4.3 = 2.3×. In order to create the need of looking through the magnifier and avoid the risk of looking besides the magnifier we opted for higher magnification and smaller symbol size. In the experiment, children moved the stand magnifier over the surface of a digitizer (sample rate 144 Hz; Wacom, Saitama, Japan; type 21ux) that was positioned horizontally in front of the child. The child sat on a height-adjustable chair to guarantee a comfortable working posture. An electronic sensor (coil) was placed in the center of the magnifier allowing X and Y movement dimensions to be recorded over time. A small camera (Pen camera, video resolution: 1281 × 960; video frame rate 30 FPS) was mounted inside the magnifier in order to record the eye during fixation (magnifier camera; **Figure 1A**). The magnifier-mounted camera did not interfere with children’s view through the magnifier. A camera placed in front of the child recorded their performance during the entire task (task-camera).

The digitizer displayed a start circle (Ø 60 mm), a large symbol (60 mm × 60 mm), a target for a matching response at the left side of the screen (match area; icon of a happy face; Ø 50 mm) and a target for a non-matching response at the right side of the screen (non-match area; icon of a sad face; Ø 50 mm; see **Figure 1B**). The large symbol was one of the LH symbols: square, circle, house, and apple (Hyvarinen et al., 1980) A sheet with printed LH symbols three steps below threshold was placed on top of the digitizer. We presented the symbols three LogMAR steps below *M*-value threshold so that children had to use the magnifier. In this way, the symbols were small enough to prevent children from seeing the symbols with their bare eyes, but large enough to identify them with the magnifier. Before the experiment started, we tested if the children were able to see the small symbols with the magnifier. We adjusted the task to their individual visual acuity to ensure equal difficulty for visually impaired and normally sighted children. The symbols were positioned at seven locations in an arc on top of the screen to allow the same distance (175 mm) between start position and symbol for all symbols (see **Figure 1B**). Except for the location of the symbols the sheet was transparent so that children were able to see the elements displayed by the digitizer (start circle, large symbol, match area; and non-match area).

The child had to place the magnifier at the start position and look at the large symbol. After 5 s the task started with an auditory signal and one of the small printed LH symbols was highlighted by a circle (target symbol; see **Figure 1B**). The child was instructed to move the magnifier as quickly as possible to the encircled symbol and then identify the symbol with the magnifier. In order to do so the child had to move head and eye in the right position. If the identified symbol was the same as the large matching symbol in the middle of the screen, the child had to move the magnifier to the match area. If the identified symbol differed from the large symbol, the child had to move the magnifier to the non-match area. This was repeated six times until all seven symbols were identified. Symbols were randomly presented. All children were given seven practice trials for the experiment started.

### Data Analysis

**FIGURE 2:**
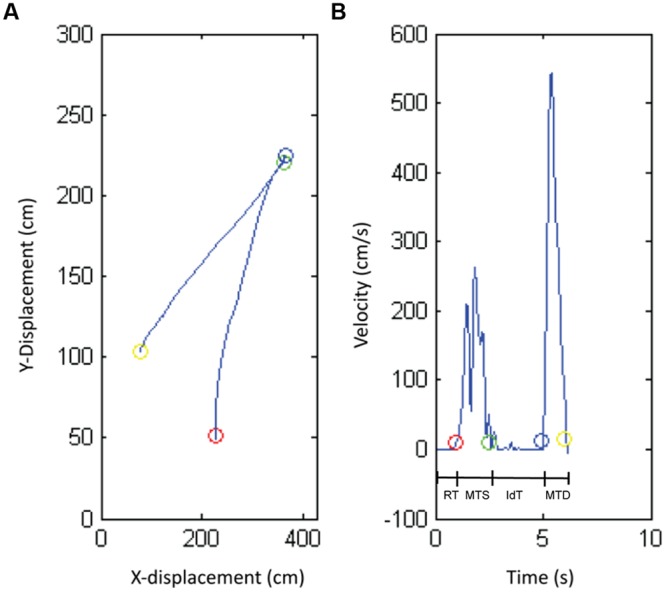
**The position **(A)** and velocity profile **(B)** produced by one representative child with normal vision.** The circles show the transitions between the different phases of the task: reaction time (RT), movement time symbol (MTS), identification time (IdT), and movement time decision (MTD).

Object position data were filtered (low-pass Butterworth filter, cut-off frequency 6 Hz; Meulenbroek et al., 2001). For each trial, success rate, reaction time (RT), movement time symbol (MTS), identification time (IdT), and movement time decision (MTD) were calculated based on position and velocity data (see **Figure 2**). Success rate is defined as the percentage of correct responses of the total number of responses. A response was “correct” when the child moved the magnifier to the match area in case of matching symbols or when the child moved the magnifier to the non-match area in case of non-matching symbols. The start and end of RT, MTS, IdT, and MTD were calculated by the moment the magnifier’s velocity exceeded (start movement) or fell below (end movement) 10 mm/s and the magnifier’s position was inside or outside relevant areas (start circle, symbol circle, match area, or non-match area). RT was the time the child needed to start the movement, defined as the time between the start of the trial and the start of the child’s movement. MTS was the time the child needed to move the magnifier from the start position to the target symbol. IdT was the time the child needed to identify the target symbol, which was the time the magnifier stayed still at the symbol. MTD was the time the child needed to move the magnifier from the symbol to the response area (match or non-match area).

In addition to these performance measures, video-recordings of each trial were made in order to obtain objective measures of the distance between child and magnifier (lens-to-magnifier distance) and eye and hand use during the task. Video-recordings of the magnifier and task cameras were synchronized with the object position data. Position data established the three phases of the task (MTS, IdT, and MTD). Two independent raters estimated viewing distance in centimeters using the height of the magnifier (6 cm) and hand width (10 cm) as indicators. Inter-rater reliability was determined with Cohen’s kappa (Huurneman et al., 2013). The raters also determined hand dominance (right, left, bimanual, or bimanual sequential, i.e., child switches hand) during the three phases of the task. Video-recordings of the camera in the magnifier were made to determine task-specific eye dominance: the eye with which the child looked through the magnifier (right or left) at the moment of identification. Two independent raters established the eye the child used during IdT in every trial.

Several tests were used to assess children’s general hand and eye dominance. Hand dominance tests were based on items of the Edinburgh Inventory (Oldfield, 1971). Children were asked to write, draw, use a spoon, throw a ball, and cut a piece of paper. Each item was assessed three times. To test eye dominance children were asked to look through an ocular and through a piece of paper with a hole in the middle (Dolman method/hole-in-the-card test). Each item was assessed two times.

For each time variable, the data from each trial was entered into Statistical Package for the Social Sciences (SPSS). Success rate per child was also entered into SPSS. An arcsine-transformation was applied to allow general linear model procedures. An analysis of variance (ANOVA) was conducted for all dependent variables with vision group as a between-subjects factor, age as a covariate, and trial as a within-subjects factor. Alpha was set at 0.05 and least-significant difference (LSD) correction was used. If age effects were significant, additional Pearson correlations were computed per vision group. Pearson correlations were computed for the associations of viewing distance with IdT and IdT with success rate. Test and task dominance were compared between groups with the nonparametric Wilcoxon signed-rank test because of unequal variances and skewed distributions. Pearson correlations were computed to assess the impact of dominant hand use on the movement time and success rate.

## Results

The results regarding performance in terms of success rate, reaction, movement, and IdT are presented below. Success rate is defined as the percentage of correct responses of the total number of responses. RT is defined as the time between the start of the trial and the start of the child’s movement. MTS was the time the child needed to move the magnifier from the start position to the target symbol. IdT was the time the child needed to identify the target symbol. MTD was the time the child needed to move the magnifier from the symbol to the response area (see **Figure 2**).

### Success Rate

For both children with visual impairment and children with normal sight, success rate differed significantly from chance (50%), *t*(28) = 8.73, *p* < 0.001, and *t*(46) = 10.93, *p* < 0.001, respectively. There was no significant difference in success rate between the visually impaired group (*M* = 80%, *SD* = 50%) and the normally sighted group (*M* = 84%, *SD* = 52%), *F*(1,75) = 0.048, *p* = 0.828.

### Reaction and Movement Times

**FIGURE 3:**
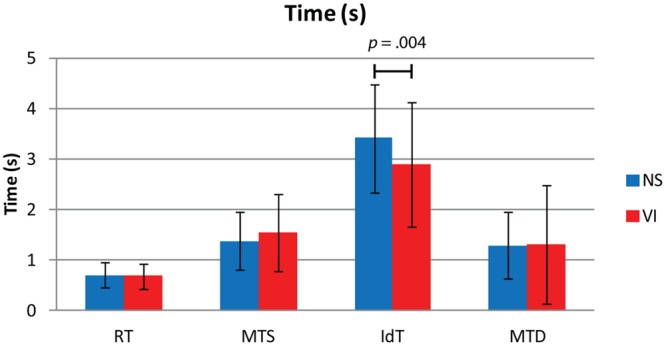
**Mean and standard deviations of reaction time (RT), movement time symbol (MTS), identification time (IdT), and movement time decision (MTD) in seconds for visually impaired (VI) and normally sighted (NS) children.** The figure depicts the *p*-value of the significant difference between normally sighted and visually impaired children for IdT.

**FIGURE 4:**
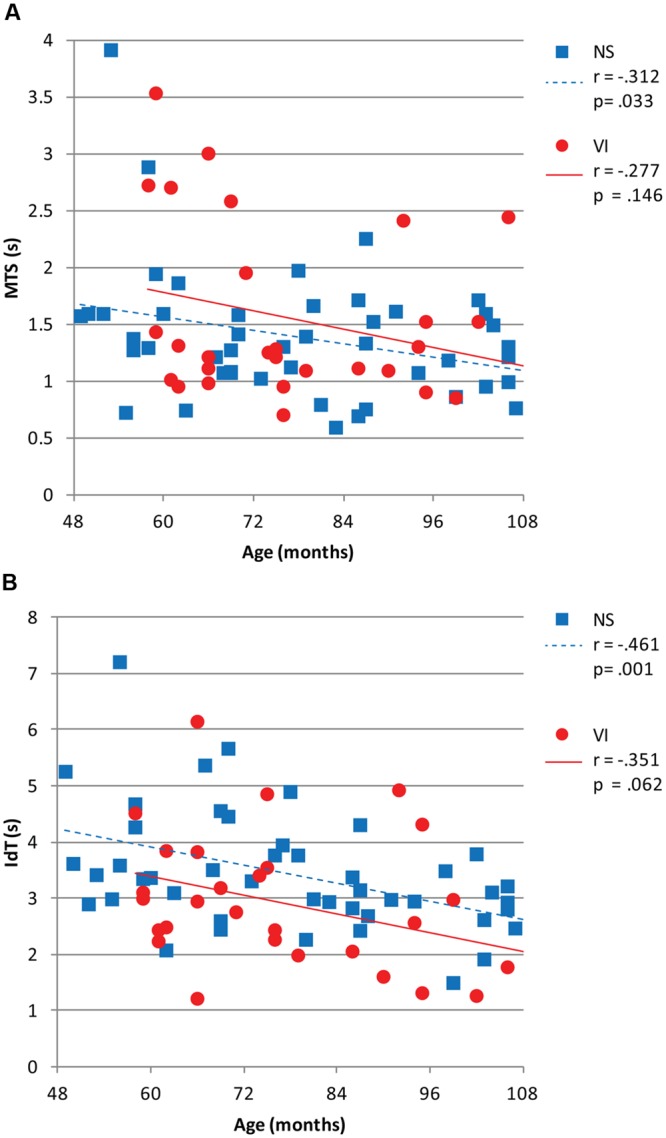
**(A)** Mean movement time symbol (MTS) and **(B)** mean identification time (IdT) in seconds plotted as a function of age in months for visually impaired and normally sighted children. Corresponding Pearson’s *r* and *p*-values are depicted.

**Figure 3** shows reaction and movement times. There were no significant differences between visually impaired and normally sighted children for RT, *F*(1,73) = 0.32, *p* = 0.858, MTS, *F*(1,73) = 1.43, *p* = 0.237, and MTD, *F*(1,73) = 1.06, *p* = 0.317. The effect of age approached significance for MTS, *F*(1,73) = 3.63, *p* = 0.062. There was no association between movement time and age in the visually impaired group, *r* = -0.28, *p* = 0.146, but a significant association in the normally sighted group, *r* = -0.31, *p* = 0.033, indicating faster performance with increasing age in the normally sighted group (**Figure 4A**).

### Identification Time

There was a significant group difference for IdT, *F*(1,73) = 9.09, *p* = 0.004, Cohen’s *d* = -0.43. Visually impaired children (*M* = 2.9 s) required less time to identify the small symbols than normally sighted children (*M* = 3.4 s; **Figure 3**). There was an age effect for IdT, *F*(1,73) = 9.91, *p* = 0.003 (**Figure 4B**), indicating faster identification of symbols with increasing age. There was a significant correlation between IdT and age in the normally sighted group, *r* = -0.46, *p* = 0.001, and a trend in the visually impaired group, *r* = -0.35, *p* = 0.062 (**Figure 4B**).

**FIGURE 5:**
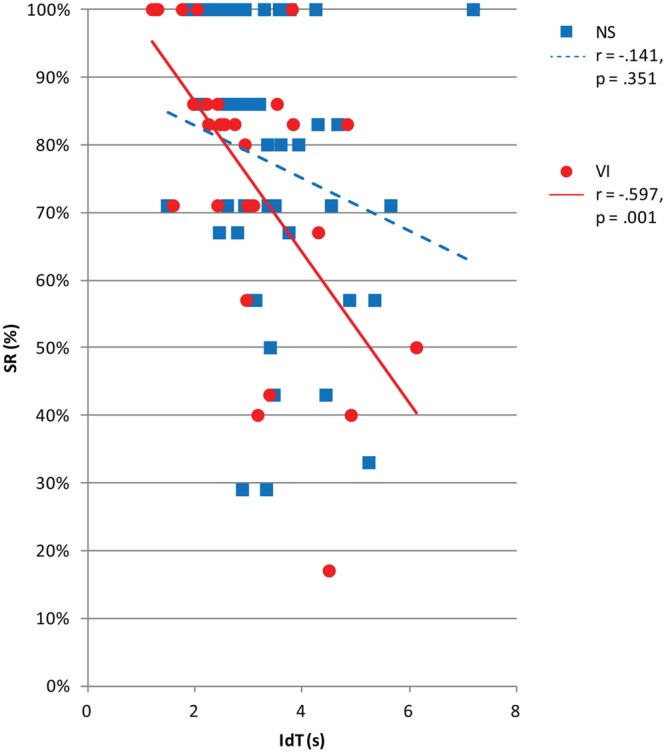
**Success rate (SR) by vision group (normally sighted, NS; visually impaired, VI) plotted as a function of identification time (IdT) in seconds.** The legend shows Pearson correlations (*r*) and corresponding *p*-values between IdT and viewing distance for each vision group.

**Figure 5** shows success rate in relation to IdT. There was a significant and moderate correlation between success rate and IdT, controlled for age, in the visually impaired group, *r* = -0.58, *p* = 0.001, but no significant correlation in the normally sighted group, *r* = -0.15, *p* = 0.332. In the visually impaired group, a shorter IdT was associated with a better success rate.

### Viewing Distance

**FIGURE 6:**
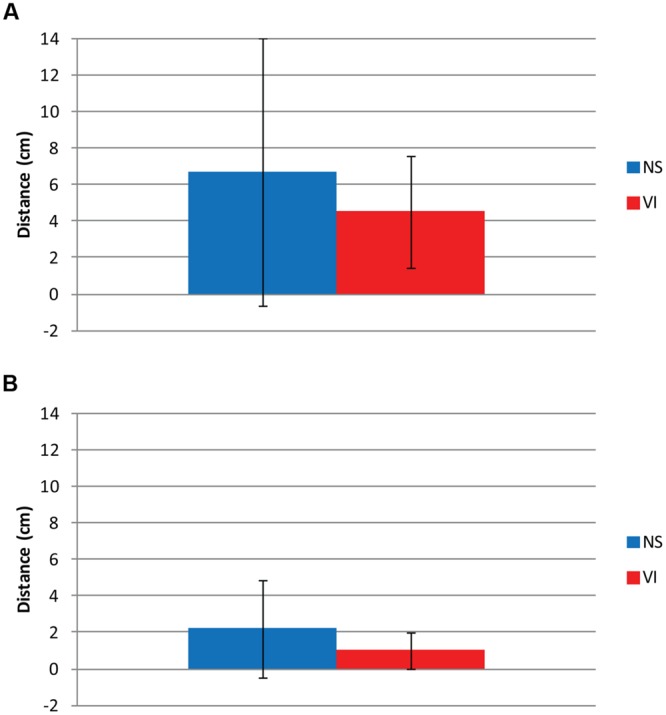
**(A)** Inter- and **(B)** intra-individual differences in viewing distance for visually impaired (VI) and normally sighted (NS) children. The inter-individual variation is the standard deviation of the mean viewing distances of all members of a group. The intra-individual variation is the mean of the group members’ standard deviations of the viewing distance over all trials for each child. **(A)** Mean viewing distance (column) and standard deviation (error-bars) between all children of the group. **(B)** Mean standard deviation over seven trails (column) and standard deviation (error-bars) of viewing distance within children with VI and NS.

**Figure 6** shows variation in viewing distance: between children (inter-individual variation, **Figure 6A**) and within children between trials (intra-individual variation, **Figure 6B**), for each group. Inter-individual variation was defined as the standard deviation of the mean viewing distances of all group members. Intra-individual variation was defined as the mean of the group members’ standard deviations of the viewing distance over all trials for each child. There was no significant group difference in mean viewing distance, *F*(1,75) = 2.81, *p* = 0.098, Cohen’s *d* = 0.45. The inter-individual variance in viewing distance (see the error-bars in **Figure 6A**) was smaller in the visually impaired group (*SD* = 2.7 cm) than in the normally sighted group (*SD* = 7.4 cm). The intra-individual variance in viewing distance (see **Figure 6B**) was smaller in the visually impaired group (*SD* = 0.9 cm) than in the normally sighted group (*SD* = 2.2 cm), *F*(1,75) = 5.83, *p* = 0.018. In the visually impaired group, *t*(28) = -9.18, *p* < 0.001, and the normally sighted group, *t*(46) = -13.22, *p* < 0.001, the intra-individual variance in viewing distance was smaller than inter-individual variance.

**FIGURE 7:**
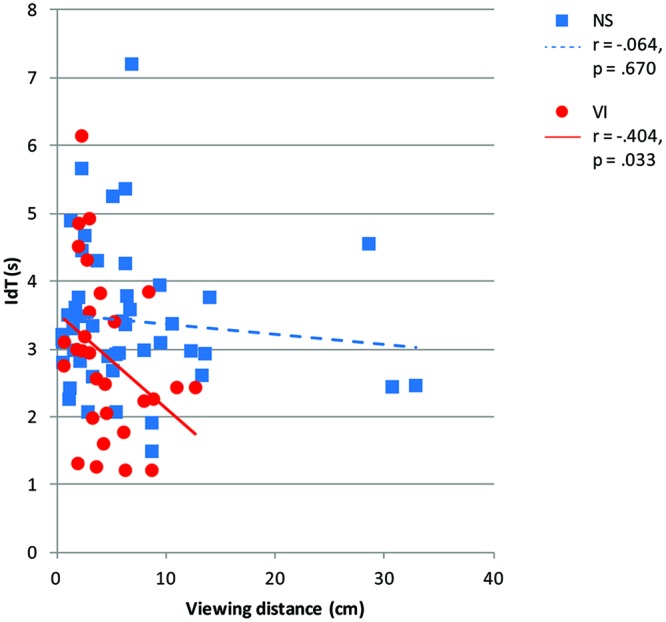
**Identification time (IdT) in seconds by vision group (normally sighted, NS; visually impaired, VI) plotted as a function of viewing distance (centimeter).** The legend shows Pearson correlations (*r*) and corresponding *p*-values between IdT and viewing distance per vision group.

**Figure 7** shows viewing distance in relation to IdT. There was a significant and moderate correlation between viewing distance and IdT controlled for age in the visually impaired group, *r* = -0.40, *p* = 0.033, but not in the normally sighted group, *r* = -0.06, *p* = 0.670. When visually impaired children chose a larger viewing distance they needed less time to identify the symbols.

### Hand and Eye Dominance

**Table 2** shows average hand and eye dominance during the magnifier task measured with hand and eye dominance tests, respectively. Eye dominance was scored per trial for 27 visually impaired children and 45 normally sighted children. Due to technical problems with the camera, the data were missing for two visually impaired and two normally sighted children. There was no meaningful difference in eye dominance during the eye dominance tests (*M* = 100%, *SD* = 0.0%) and the magnifier task (*M* = 98.1%, *SD* = 7.8%) in the normally sighted group, *Z* = -1.08, *p* = 0.276. There also was no meaningful difference in eye dominance during the eye dominance tests (*M* = 98.2%, *SD* = 6.7%) and the magnifier task (*M* = 99.5%, *SD* = 2.7%) in the visually impaired group, *Z* = -1.45, *p* = 0.655. There was no meaningful difference in hand dominance during the hand dominance tests (*M* = 99.75%, *SD* = 1.3%) and the magnifier task (*M* = 97.3%, *SD* = 6.9%) in the visually impaired group, *Z* = -1.79, *p* = 0.074. In the normally sighted group, hand dominance was higher in the hand dominance tests (*M* = 99.9%, *SD* = 1.0%) than in the magnifier task (*M* = 92.7%, *SD* = 13.2%), *Z* = -3.39, *p* = 0.001. Within the groups, there was no significant relation between percentage of hand dominance and movement time or success rate.

**Table 2:** Mean hand and eye dominance of visually impaired and normally sighted children during the magnifier task and dominance tests.

	Magnifier task			Hand dominance tests	
	Right hand	Left hand	Bimanual	Right hand	Left hand
VI	19	5	5	23	5
NS	34	5	8	43	4

	**Magnifier task**			**Eye dominance tests**	
	**OD**		**OS**	**OD**	**OS**

VI	13		14	10	17
NS	26		19	30	17

*VI, visually impaired children; NS, normally sighted children; OD, right eye; OS, left eye.*

## Discussion

In this study, effectiveness and efficiency of magnifier use in children with visually impairment was investigated in an ecologically valid goal-directed perceptuomotor task. Both children with visual impairment (mean success rate: 80%) and children with normal sight (mean success rate: 84%) could adequately identify the symbols with the stand magnifier. Visually impaired children’s performance in terms of success rate, mean reaction time, and mean movement time of first and second movement parts, did not differ from normally sighted children. In contrast to our hypothesis, children with visual impairment required less time to identify small symbols with a stand magnifier than children with normal vision. The variation in viewing distance between trials for each child and between children within each vision group was smaller in the visually impaired group than in the normally sighted group. In the visually impaired group, a larger viewing distance was associated with a shorter identification time, which in turn was associated with a higher success rate.

Thus, visually impaired children were able to perform the task with the stand magnifier as adequately and successfully as normally sighted children. To draw a fair comparison, we ensured equal difficulty for all children by adjusting the symbol size to individual visual acuity, that is, three steps below the individually established threshold acuity for each child.

Nevertheless, one might argue that the stand magnifier is not the most obvious choice in young children because of its large magnification and monocular use. In normally sighted children (6–14 years) and adults, binocular acuity is better than monocular acuity (Vedamurthy et al., 2007), a phenomenon called binocular summation (Blake and Fox, 1973). Due to the development of visual acuity of the dominant eye, in normal development the binocular summation ratio decreases with age (Vedamurthy et al., 2007). Although there is considerable ambiguity regarding monocular compared to binocular viewing in normally sighted children and children with visual impairment in this task (Vedamurthy et al., 2007; Huurneman and Boonstra, 2013), children were perfectly able to adopt to the stand magnifier with monocular requirements. Furthermore, both visually impaired and normally sighted children in the present study already had a dominant eye that they used to look through the magnifier and identify the symbol. Similar to the reasoning in several previous studies (Cox et al., 2009; Reimer et al., 2011; Boonstra et al., 2012), this made the stand magnifier a suitable tool for young children with visual impairment for the present task.

Efficiency of children’s performance with a magnifier was investigated in two phases. First, this study investigated the efficiency of children’s movement with the magnifier to the target area. We hypothesized that visually impaired children needed more time than normally sighted children in Phase 1, because goal-directed movements with a cylinder object are less well developed in children with visual impairment than in children with normal vision (Reimer et al., 2008; Liebrand-Schurink et al., 2015). This hypothesis was not confirmed, because visually impaired children were able to handle the stand magnifier according to the task requirements at approximately the same speed as normally sighted children did. The minor difference between the groups might be explained by the difference in variation in hand use. The normally sighted children showed more variation in which hand they used to manipulate the magnifier compared to the pre-test of hand dominance. The visually impaired children primarily used their preferred hand. A combination of factors (Leconte and Fagard, 2004; Cox and Smitsman, 2006a,b; Streri and de Hevia, 2014) influence children’s hand selection, such as handedness (Hopkins and Rönnqvist, 1998), object position (Harris and Carlson, 1993; Van Hof et al., 2002), and task complexity (Bryden et al., 1999; Leconte and Fagard, 2004). Studies have shown that the preferred hand is used more frequently in complex tasks than in simple grasping tasks (Steingrueber, 1975; Leconte and Fagard, 2004). In the present study, visually impaired children may have perceived the complexity of the task as relatively high and may therefore have chosen their preferred hand, while the normally sighted children may have perceived the complexity of the task as relatively low, and therefore used their preferred hand less frequently. However, in the present study *post hoc* tests evaluating hand dominance effects did not reveal any effect. Thus although the manual preference found in this complex tasks is in accordance with literature reports (e.g., Leconte and Fagard, 2004), this motor control aspect of LVA use clearly needs further investigation in a more specific experimental setup.

Second, this study investigated the efficiency of symbol identification with a stand magnifier. Children with visual impairment were even more efficient than children with normal vision in identifying small symbols. We expected that visually impaired children would require more time to identify small symbols than normally sighted children, because they have less experience with small details, but the opposite was found. A possible explanation for this result might be found in the strategies that were performed for symbol identification. This is discussed below in relation to viewing distance and age-related changes.

We can conclude that both visually impaired and normally sighted children shorten their viewing distance. Young children are used to accommodate when stimulated with tiny details (the eyes adjust fixation from one point in space to another). This accommodative response is strong and is performed together with convergence (realignment; Bharadwaj and Candy, 2008). At a distance of about 4 cm this strong accommodative response is needed. The combination of this response with monocular viewing is not easy for a child with good binocular vision. However, it is possible that young children are used to respond with accommodation and use this reflex for a short distance if needed, for instance for a specific magnifier. Previous research has shown that in young children accommodative gain is reduced during monocular viewing relative to binocular viewing and reaches adults levels at the age of 7–10 years (Bharadwaj and Candy, 2008). The typically developing visual system compensates for temporarily induced conflicts between blur and disparity, without exhibiting a strong preference for either cue. The accuracy of this compensation decreases with an increase in amplitude of cue-conflict (Bharadwaj and Candy, 2009). In our study, the cue conflict was large due to the magnifier, offering blur and aniseikonia (difference in retinal image size between the eyes). The response can be a purposeful suppression of one image while accommodating on the other; this might be a very difficult binocular task for children. In typically developing children (3.1 months to 12.1 years) induced aniseikonia (by placing a 11% afocal magnifier to the right eye) did not significantly influence the gain of accommodation and vergence (Bharadwaj and Candy, 2011), but the effect of aniseikonia on visually impaired children is still unclear. Magnifier use was compared to enlarged print (Huurneman et al., 2013), but in this static task there was no cue conflict between the two eyes because they used a large dome magnifier enabling children to look at the symbols binocularly and perception of the surroundings was not relevant. Quantitative and qualitative performance of magnifier use has been assessed in visually impaired children in a dynamical trail-following task (Cox et al., 2009). In relation to this task, viewing behavior was assessed (Boonstra et al., 2012). The viewing distance on near visual acuity assessment was measured before and after the training. After the training the children significantly reduced their viewing distance from 9.5 to 7.9 cm on the LH near vision test single, and they reduced their viewing distance from 10.0 to 7.6 cm on the LH near vision line. However, the children in the control group, that performed the trail-following task without a magnifier, demonstrated the same reduction of viewing distance. The authors argue that reduction of the viewing distance during near visual acuity assessment is probably a “spin-off” of the intensive visual attention applied during the trail-following game.

In our study, the variation in viewing distance over trials between children and within children was smaller for the visually impaired group than for the normally sighted group. In the visually impaired group, a larger viewing distance was associated with faster identification, and faster identification was associated with better performance (i.e., more correct answers). This a strategy with less variation in viewing distance that resulted in efficient magnifier use. In this specific task, this strategy is efficient because at a short distance children can use the same accommodative level during identification.

Normally sighted children identified the symbols more slowly than visually impaired children did. In this respect, the larger variation in viewing distance between children (inter-individual variance) and between trials within a child (intra-individual variance) in the normally sighted group indicates that different strategies for identifying symbols were explored. Normally sighted children might make better use of a variation in their natural accommodation range but also to shorten their viewing distance. This strategy leads to an alternation of distance to the magnifier from trial to trial in normally sighted children. Although not investigated in this study, such an explorative strategy may indicate a learning process (Braun et al., 2009) which may have lead (temporarily) to slower identification in this task. In the long-term, however, this exploration and the associated motor learning might be highly beneficial, resulting in more adaptive and flexible viewing behavior in normally sighted children. This learning curve hypothesis is supported by the finding that in normally sighted children, but not in visually impaired children, faster identification was associated with increasing age.

This study demonstrated that the stand magnifier is a suitable tool for young visually impaired children in an ecologically valid task. The findings suggest that visually impaired children choose a standard but less adaptive strategy in which they primarily used their preferred hand to manipulate the magnifier and their preferred eye to identify the symbol. How this might influence the development of their viewing behavior is an issue that deserves further investigation.

## Author Contributions

JL-S made substantial contributions to the conception and design of the work, the acquisition, analysis, and interpretation of data for the work and drafted the work critically for important intellectual content. RC made substantial contributions to the conception and design of the work, the analysis, and interpretation of data for the work and revised the work critically for important intellectual content. GR, AC, and RM made substantial contributions to the conception of the work, the interpretation of data for the work and revised the work critically for important intellectual content. FB made substantial contributions to the conception and design of the work; the acquisition and interpretation of data for the work and revised the work critically for important intellectual content. All authors agree to approve the version to be published and to be accountable for all aspects of the work in ensuring that questions related to the accuracy or integrity of any part of the work are appropriately investigated and resolved.

## Conflict of Interest Statement

The authors declare that the research was conducted in the absence of any commercial or financial relationships that could be construed as a potential conflict of interest.
